# The Standard-Dose Heparin-Warfarin Remedy Partially Resolves Thrombi in the Right Superior Pulmonary Vein and Left Atrium and Ameliorates Type 2 Diabetes Mellitus

**DOI:** 10.7759/cureus.57323

**Published:** 2024-03-31

**Authors:** Hidekazu Takeuchi

**Affiliations:** 1 Internal Medicine and Cardiology, Takeuchi Naika Clinic, Ogachi-Gun, JPN

**Keywords:** tee, 80-mdct, warfarin, heparin, pulmonary vein thrombi, pulmonary vein thrombosis, t2dm

## Abstract

Pulmonary vein thrombosis is common and underdiagnosed. Previously, we reported several cases of pulmonary vein thrombi (PVTs) using cardiac computed tomography (CT) and transesophageal echocardiography (TEE).

We reported that warfarin and direct oral anticoagulants (DOACs) partially resolved PVTs; however, it is difficult to resolve all PVTs completely. Therefore, we evaluated the effects of standard-dose heparin-warfarin remedy on PVTs and left atrium (LA) thrombi using TEE and cardiac CT.

A 64-year-old male with type 2 diabetes mellitus (T2DM) and hypertension was assessed for thrombi in the LA and pulmonary veins using TEE and 80-slice multidetector computed tomography (80-MDCT).

After one month of standard-dose heparin-warfarin remedy, the patient’s right superior pulmonary vein (RSPV) thrombi and expanded LA thrombi from the RSPV thrombi had partially resolved. The RSPV thrombi and the expanded LA thrombi from the RSPV thrombi were detected using cardiac CT and TEE; however, they were depicted as black areas on TEE. They periodically moved inward with the patient’s heartbeats.

Additionally, the standard-dose heparin-warfarin remedy ameliorated the patient’s T2DM, and the remedy effect could be maintained for five months to some extent by administering a standard dose of warfarin. The standard-dose heparin-warfarin remedy can ameliorate not only T2DM but also diabetic complications such as diabetic nephropathy and gestational diabetes mellitus.

## Introduction

Pulmonary vein thrombosis is common [1]. Our previous reports showed that easily 61% of older individuals with chest pain had pulmonary vein thrombi (PVTs) [2]. In many cases, each patient had thrombi in several pulmonary veins, and these thrombi formed a network of PVTs in the left atrium (LA) [3]. We reported several cases of PVTs in older individuals with or without chest pain [1-7].

PVTs can induce acute myocardial infarction (AMI), ischemic stroke (IS) and systemic thrombosis by emitting rather large thrombi [8]. A study of retrieved thrombi in patients with AMI or IS indicated that the retrieved thrombi had calcification [9,10], suggesting that the thrombi were old. Therefore, these patients are likely to have thrombi before AMI or IS occurs. Our previous reports showed that the body can have old thrombi in the pulmonary vein and the LA. Moreover, PVTs can emit smaller particles, including neutrophil extracellular traps (NETs), and other components, such as DNA and histones. Currently, NETs are known to be associated with several diseases, such as type 2 diabetes mellitus (T2DM) [11,12], atherosclerosis, diabetic nephropathy [13], diabetic retinopathy [14] and gestational diabetes mellitus [15].

During pulmonary infection, NETs destroy microorganisms and promote arterial thrombus (AT) formation to inhibit the spread of pathogens to all organs through the pulmonary vein. Then, ATs in the pulmonary vein increase in size and length. Our previous report showed that small PVTs nurtured larger in the larger pulmonary vein [7], expanded into the LA and adhered to the wall of the LA [4-6]. These patients had right inferior pulmonary vein (RIPV) thrombi identified via cardiac computed tomography (CT) and transesophageal echocardiography (TEE) [16]. Expanded LA thrombi from the RIPV thrombi are occasionally not detected using cardiac CT, which is the most important feature of expanded LA thrombi from the RIPV [4,6].

According to our data, it might be difficult to completely resolve PVTs using warfarin or direct oral anticoagulants (DOACs) [2,5,16]. Heparin is known to break up histones in NETs [17,18]. Therefore, we evaluated the effects of standard-dose heparin-warfarin remedy on PVTs using 80-slice multidetector computed tomography (80-MDCT) and TEE, and we evaluated changes in blood glucose levels.

## Case presentation

A 64-year-old male with T2DM and hypertension was assessed for thrombi in the LA and pulmonary veins using TEE and 80-MDCT. The patient was treated for diabetes mellitus with sitagliptin (50 mg; once a day) and pioglitazone (30 mg; once a day), and his hypertension was treated with olmesartan (20 mg; once a day) and amlodipine (2.5 mg; once a day). The patient took these four medications after breakfast. The patient had no symptoms of chest pain, fever, cough, sputum or IS. The respiratory exam did not show decreased breath sounds, lung crackles or wheezing. The cardiac exam did not show a heart murmur or arrhythmia. His height was 168 cm, and his weight was 72 kg. A chest roentgenogram indicated no lung cancer or cardiomegaly. No previous remedy with warfarin or DOACs was given. ECG indicated sinus rhythm, a normal axis and no ST-T changes, and the patient’s heart rate was 68 beats/min. The serum D-dimer level was 0.7 μg/ml (normal; < 1.0 μg/ml), the activity of protein S was 105% (normal; 74-132%), and the activity of protein C was 102% (normal; 64-135%). The homocysteine level was 12.0 nmol/mL (normal; 5-15 nmol/mL). His C-reactive protein (CRP) level was 0.18 mg/dL (normal; 0.00-0.19 mg/dL). His glycosylated hemoglobin (HbA1c) level was 7.1%.

**Figure 1:**
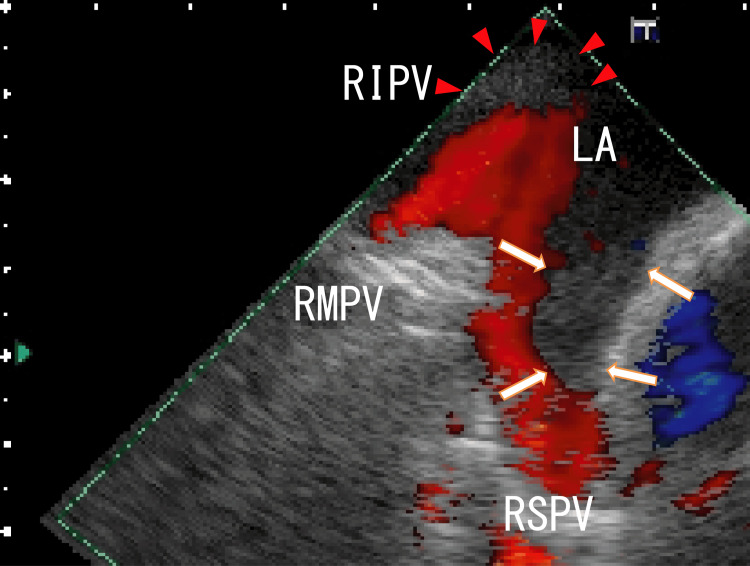
TEE images showing thrombi in the left atrium Transesophageal echocardiography (TEE) images showing expanded left atrium (LA) thrombi from the right superior pulmonary vein (RSPV), and these thrombi were dark masses that appeared to be adhered to the wall of the RSPV and the LA. Thrombi were identified as a lack of blood flow from the RSPV (arrows). The thrombi had no white areas at all. Blood flows from the RSPV and right middle pulmonary vein (RMPV) were shown as red areas. Thrombi expanded from the right inferior pulmonary vein (RIPV) and were depicted as dim white areas (arrowheads). Left atrium diverticula (LADs) were not detected.

TEE showed thrombi in the LA that were slightly whitish, and these thrombi expanded from the right superior pulmonary vein (RSPV) and appeared to attach to the anterosuperior wall of the LA around the exit of the RSPV (Figure 1). The LA thrombi had periodical movement with the patient’s heartbeats (Video 1). The thrombi had no hyperechoic areas that had been observed in our previous cases [3,5].

**Video 1:** TEE images showing thrombi in the left atrium Transesophageal echocardiography (TEE) images showing expanded left atrium (LA) thrombi from the right superior pulmonary vein (RSPV), and the thrombi appears as a dark mass that appears to be adhered to the wall of the RSPV and the LA. Thrombi were detected due to the lack of blood flow from the RSPV. The LA thrombi moved with the heartbeats. The blood flows from the RSPV and right middle pulmonary vein (RMPV) are shown as red areas. Blood flow from the right inferior pulmonary vein (RIPV) could not be detected; however, white LA thrombi were observed in front of the exit of the RIPV and did not move with the heartbeats. The thrombi were likely the expanded LA thrombi from the RIPV thrombi. Left atrium diverticula (LADs) were not detected.

**Figure 2:**
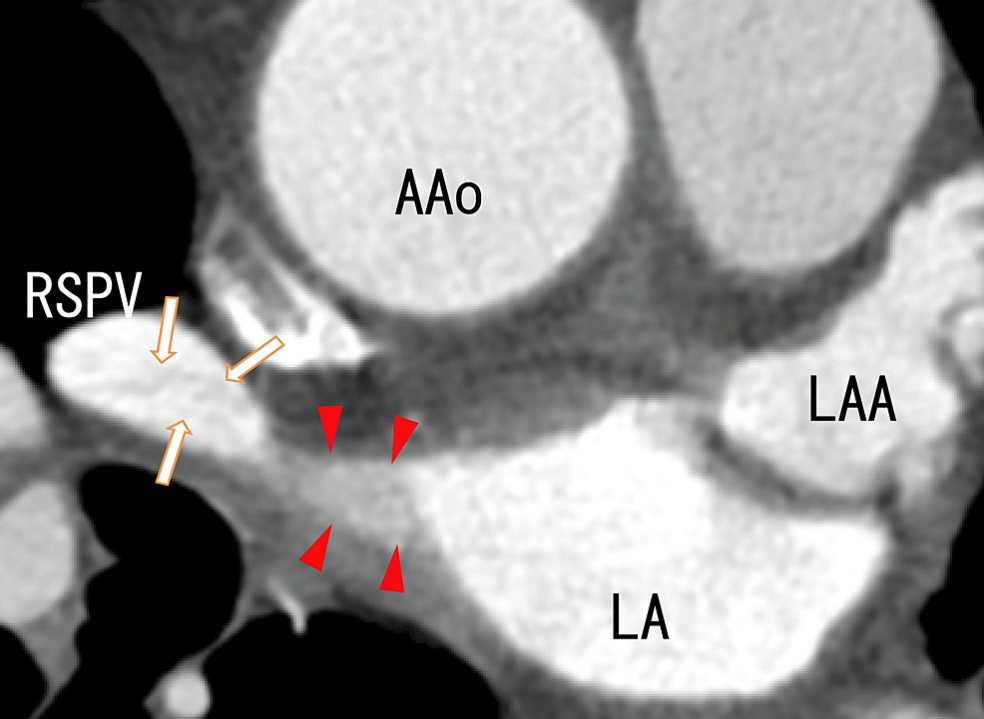
Axial images from an 80-MDCT scan showing thrombi in the RSPV and LA Axial images from an 80-slice multidetector computed tomography (80-MDCT) scan showing the right superior pulmonary vein (RSPV) and left atrium (LA). There were rather dark areas in the RSPV (arrows), suggesting that there were thrombi in the RSPV. There were dark areas in the left atrium (arrowheads), suggesting that there were thrombi in the left atrium. No left atrium diverticula (LADs) were detected. AAo, ascending aorta; LAA, left atrium appendage

**Figure 3:**
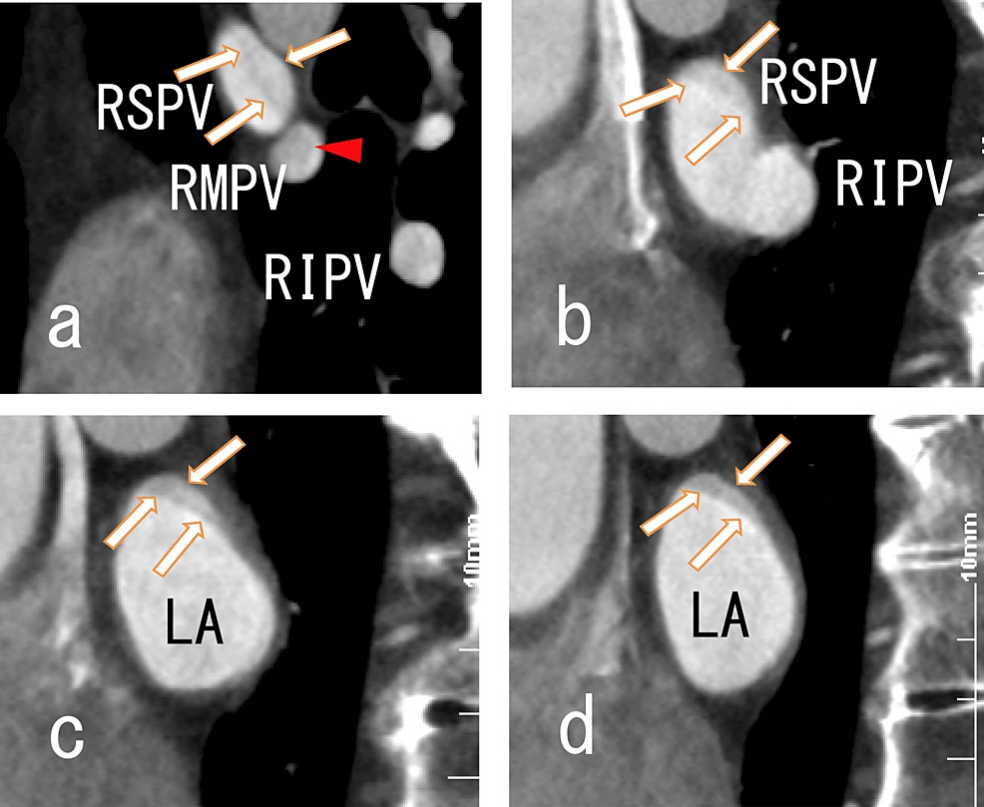
Sagittal images from an 80-MDCT scan showing thrombi in the RSPV and LA Sagittal images from an 80-MDCT scan showing the RSPV, RMPV, RIPV and right side of the LA. There were some images of thrombi in the RSPV, RMPV, RIPV and LA. No LADs were identified. (a) There were rather dark areas in the RSPV (arrows), suggesting that there were thrombi in the RSPV. There were rather dark areas in the RMPV (arrowhead), suggesting that there were thrombi in the RMPV. There were dim dark areas in the RIPV. (b) After the RSPV’s connection to the RMPV, the RSPV then linked to the RIPV. There were rather dark areas in the posterosuperior region of the RSPV (arrows), suggesting that there were thrombi in the RSPV. (c) and (d) There were rather dark areas in the posterosuperior region of the LA (arrows), suggesting that there were thrombi in the LA. The thrombi were adhered to the posterosuperior wall of the LA. 80-MCDT, 80-slice multidetector computed tomography; LA, left atrium; RIPV, right inferior pulmonary vein; RMPV, right middle pulmonary vein; RSPV, right superior pulmonary vein; LADs, left atrium diverticula

80-MDCT showed dimly thinned thrombi in the RSPV and LA, suggesting thrombi in the RSPV and LA (Figures 2-3).

**Figure 4:**
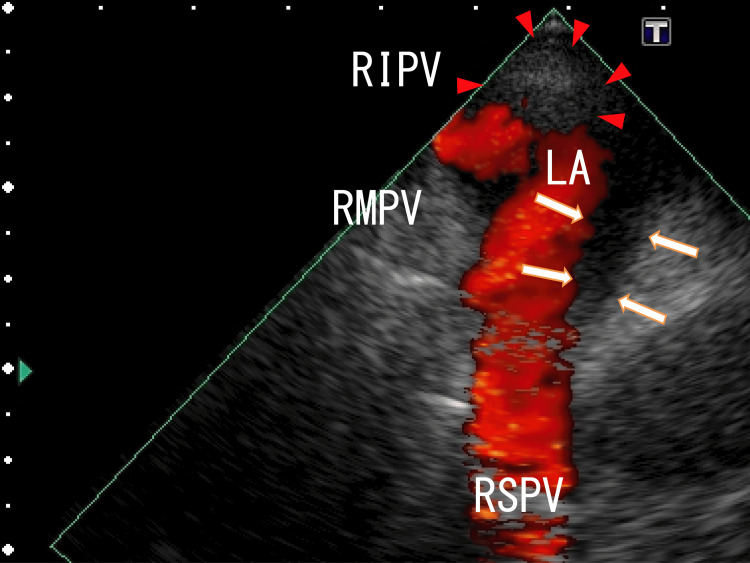
TEE images obtained after one month of standard-dose heparin-warfarin remedy showing partially resolved thrombi TEE images obtained after one month of standard-dose heparin-warfarin remedy showing partially resolved thrombi in the RSPV and LA that appeared to be linked to the thrombi in the RSPV (arrows). The thrombi included no white areas. Thrombi expanded from the RIPV and were depicted as dim white areas (arrowheads). Blood flow from the RSPV and RMPV were shown as red areas. The blood flow from the RIPV could not be detected; however, white LA thrombi were observed in front of the exit of the RIPV. The thrombi were likely the expanded LA thrombi from the RIPV thrombi. LA, left atrium; RIPV, right inferior pulmonary vein; RMPV, right middle pulmonary vein; RSPV, right superior pulmonary vein; TEE, transesophageal echocardiography

After one month of standard-dose heparin-warfarin remedy on admission, TEE showed that the LA thrombi were decreased but were present in a position similar to that before the remedy (Figure 4 and Video 2).

**Video 2:** TEE images obtained after one month of standard-dose heparin-warfarin remedy showing partially resolved thrombi TEE images obtained after one month of standard-dose heparin-warfarin remedy showing partially resolved thrombi in the RSPV and LA that appeared to be linked to the thrombi in the RSPV (arrows). The thrombi included no white areas and moved with his heartbeats. The blood flows from the RSPV and RMPV are shown as red areas. Blood flow from the RIPV could not be identified; however, white LA thrombi were observed in front of the exit of the RIPV. The thrombi were likely the expanded LA thrombi from the RIPV thrombi. The thrombi did not move with the heartbeats. TEE, transesophageal echocardiography; RIPV, right inferior pulmonary vein; RMPV, right middle pulmonary vein; RSPV, right superior pulmonary vein; LA, left atrium

**Figure 5:**
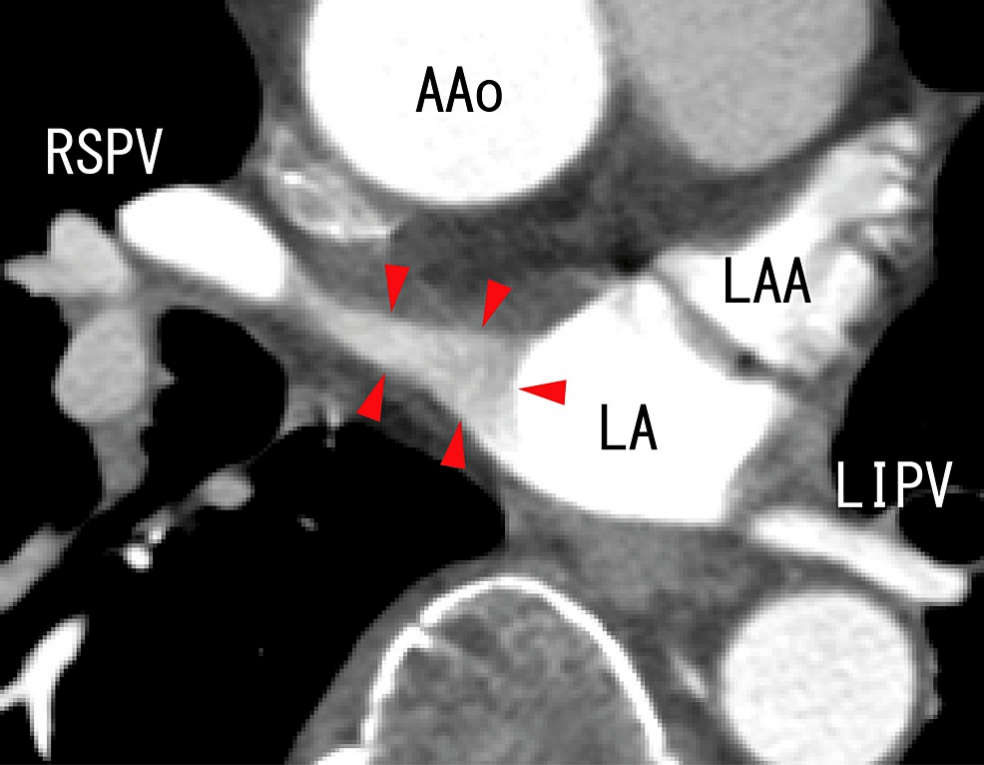
Axial images from an 80-MDCT scan obtained after one month of standard-dose heparin-warfarin remedy showing LA thrombi Axial images from an 80-MDCT scan obtained after one month of standard-dose heparin-warfarin remedy showing the RSPV, left inferior pulmonary vein (LIPV) and LA. There were no images of thrombi in the RSPV, suggesting that the thrombi in the RSPV had resolved. The dark area of the LA became slightly larger (arrowheads), suggesting that the thrombi might have become larger. However, the observed range of the LA was not necessarily the same between Figure 2 and Figure 5. AAo, ascending aorta; LA, left atrium; LAA, left atrium appendage; RSPV, right superior pulmonary vein; 80-MDCT, 80-slice multidetector computed tomography

**Figure 6:**
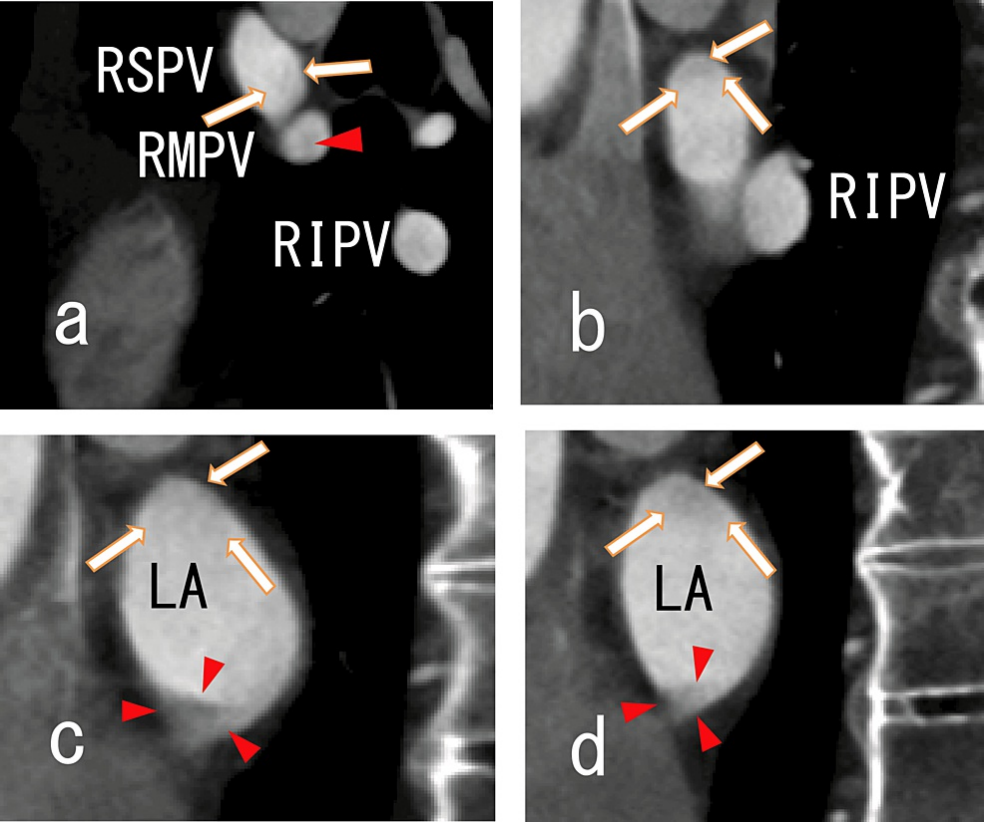
Sagittal images from an 80-MDCT scan obtained after one month of standard-dose heparin-warfarin remedy showing thrombi in the RSPV, RMPV, RIPV and LA (a) There were rather dark areas in the RSPV (arrows), suggesting that thrombi were partly resolved. There were rather dark areas in the RMPV (arrowhead), suggesting that there were thrombi in the RMPV. There were dim dark areas in the RIPV. (b) After the RSPV’s connection to the RMPV, the RSPV then linked to the RIPV. There were rather dark areas in the anterosuperior region of the RSPV (arrows), suggesting that there were thrombi in the RSPV. There were dim dark areas in the RIPV. (c) There were thin, vague, dark areas in the superior region of the LA (arrows), suggesting that the thrombi in the LA had partially resolved. There were new dark areas in the inferior region of the LA (arrowheads). (d) There were rather dark areas in the posterosuperior region of the LA (arrows), suggesting that there were thrombi in the LA. The merging of the thrombi was vague. There were new dark areas in the inferior region of the LA (arrowheads). LA, left atrium; RIPV, right inferior pulmonary vein; RMPV, right middle pulmonary vein; RSPV, right superior pulmonary vein; 80-MDCT, 80-slice multidetector computed tomography

80-MDCT showed thrombi in the RSPV and LA, which were adhered from the anterosuperior wall to the posterosuperior wall of the LA (Figures 5-6). The images showed that the thrombi became vaguer and slightly larger.

One week after the start of the remedy, the patient showed a decrease in blood glucose levels before meals compared to those in the normal range, especially at lunch, so the medication used to treat his T2DM was stopped two weeks after admission to avoid hypoglycemia. His HbA1c decreased to 6.9% at discharge despite stopping diabetes mellitus medications.

**Figure 7:**
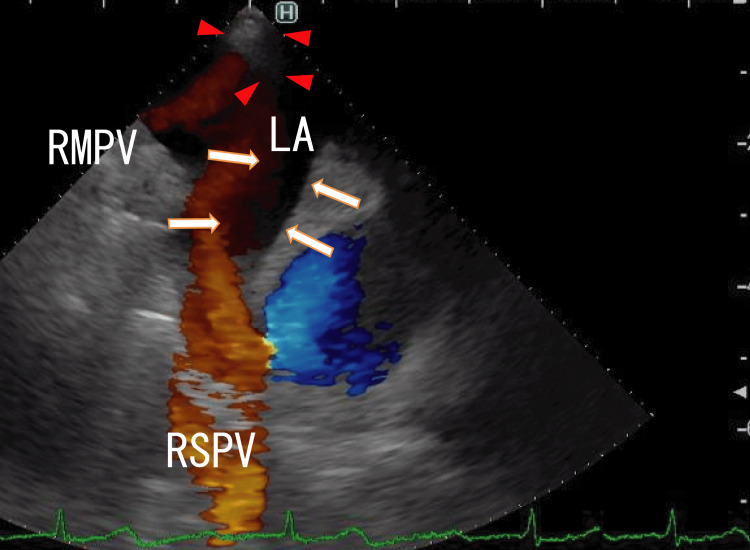
TEE images obtained after five months of standard-dose warfarin remedy showing thrombi in the LA TEE images obtained after five months of standard-dose warfarin remedy showing thrombi in the LA that appeared to be linked to thrombi in the RSPV (arrows). The thrombi included no white areas. Thrombi expanded from the RIPV and were depicted as dim white areas that looked like to be decreased (arrowheads). LA, left atrium; RMPV, right middle pulmonary vein; RSPV, right superior pulmonary vein; TEE, transesophageal echocardiography

**Figure 8:**
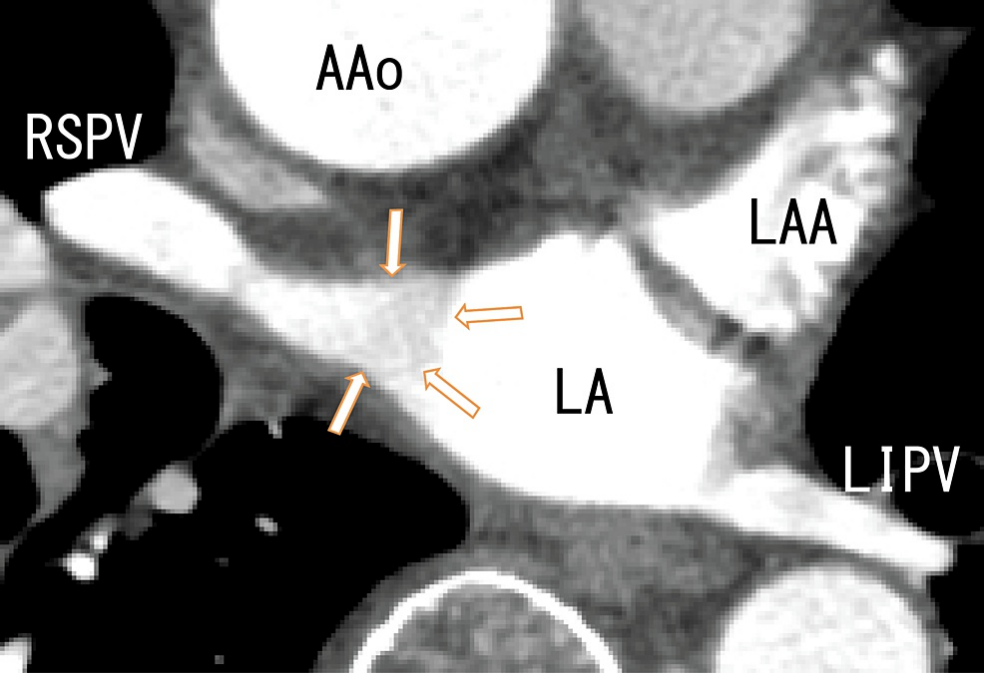
Axial images from an 80-MDCT scan obtained after five months of standard-dose warfarin remedy showing LA thrombi Axial images from an 80-MDCT scan obtained after five months of standard-dose warfarin remedy showing RSPV, LIPV and LA. There were some vague dark images in the LA (arrows), suggesting thrombi in the LA. The dark area of the RSPV became clearer, suggesting that the thrombi had resolved. AAo, ascending aorta; LAA; left atrium appendage; LA, left atrium; LIPV, left inferior pulmonary vein; RSPV, right superior pulmonary vein; 80-MDCT, 80-slice multidetector computed tomography

**Figure 9:**
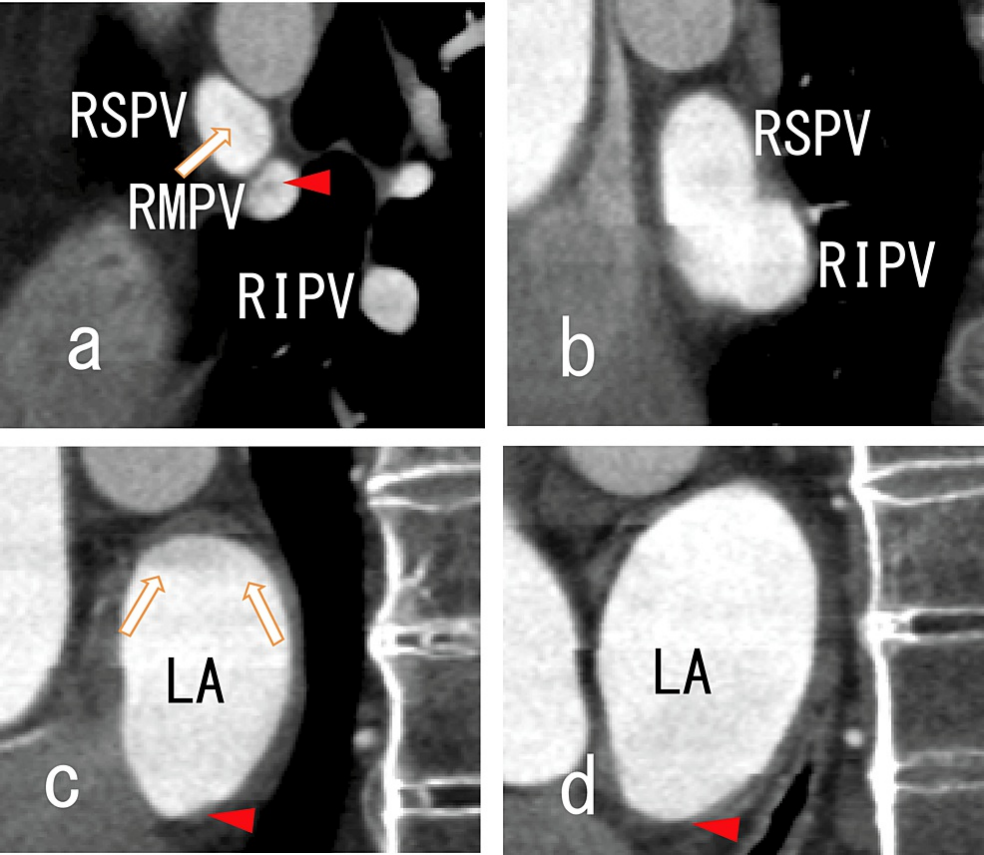
Sagittal images from an 80-MDCT scan obtained after five months of standard-dose warfarin remedy Sagittal images from an 80-MDCT scan obtained after five months of standard-dose warfarin remedy showing thrombi in the RSPV, RMPV, RIPV and LA. (a) There were rather dark areas in the RSPV (arrow), suggesting that the thrombi were partly resolved. There were rather dark areas in the RMPV (arrowhead), suggesting that there were thrombi in the RMPV. There were dim dark areas in the RIPV dimly. All the dark areas in the vessels became slightly clearer, suggesting that all the thrombi had partly resolved. (b) After the RSPV’s connection to the RMPV, the RSPV then linked to the RIPV. There were rather dim, dark areas in the RSPV and RIPV, suggesting that the thrombi had partially resolved. (c) There were thin, vague, dark areas in the superior region of the LA (arrows), suggesting that the thrombi in the LA had partially resolved. There were no new dark areas in the inferior region of the LA (arrowhead). (d) There were no dark areas in the LA. There were no new dark areas in the inferior region of the LA (arrowhead). LA, left atrium; RIPV, right inferior pulmonary vein; RMPV, right middle pulmonary vein; RSPV, right superior pulmonary vein; 80-MDCT, 80-slice multidetector computed tomography

After discharge, an additional five months of standard-dose warfarin remedy (4.5 mg once a day) was administered, and the thrombi appeared to be partially resolved and continued to resolve (Figures 7-9 and Video 3). In Video 3, some parts of the RIPV thrombi sometimes appeared and moved with breathing. The medications for T2DM were stopped because his HbA1c levels were under 7.0% for four months. Five months later, his HbA1c was 7.1% without diabetes mellitus medications. Although the patient was treated with hypertension medications, his blood pressure was not affected by standard-dose heparin-warfarin remedy.

**Video 3:** TEE images obtained after five months of standard-dose warfarin remedy showing LA thrombi TEE images obtained after five months of standard-dose warfarin remedy showed thrombi in the LA that appeared to be linked to the thrombi in the RSPV. The thrombi, including those without white areas, periodically moved inward with the patient’s heartbeats. The blood flows from the RSPV and the RMPV are shown as red areas. Blood flow from the RIPV could not be identified; however, white LA thrombi were observed in front of the exit of the RIPV. The thrombi were likely the expanded LA thrombi from the RIPV thrombi. The thrombi did not move with the heartbeats. Other parts of the RIPV thrombi sometimes appeared and moved with breathing. TEE, transesophageal echocardiography; LA, left atrium; RIPV, right inferior pulmonary vein; RMPV, right middle pulmonary vein; RSPV, right superior pulmonary vein

## Discussion

To our knowledge, the present case report is the first to show that standard-dose heparin-warfarin remedy resolved RSPV thrombi and expanded LA thrombi from RSPV thrombi and ameliorated moderate T2DM.

This is the first report of expanded LA thrombi from RSPV thrombi diagnosed using TEE; these thrombi were adhered to the anterosuperior wall of the LA and moved periodically with the heartbeats. The thrombi could be detected using 80-MDCT. We have previously reported that when LA thrombi from RIPV thrombi attach to the anterosuperior wall of the LA, the LA thrombi look much whiter and do not move periodically with the heartbeats [4,6]. However, then 80-MDCT did not detect expanded LA thrombi [4,6]. Why such differences are observed is unclear.

In the present case, there were no expanded LA thrombi from the RIPV thrombi adhering to the anterosuperior wall of the LA. Moreover, there were no left atrial diverticula (LADs), despite the attachment of the expanded LA thrombi from the RSPV. It is possible that the characteristics of RSPV thrombi are quite different from those of RIPV thrombi; at least, the color of the thrombi observed using TEE is different; and how to visualize the thrombi using 80-MDCT is different.

Treatment with standard-dose heparin-warfarin for one month partially resolved the thrombi in the RSPV, right middle pulmonary vein (RMPV), and RIPV, as determined by 80-MDCT, and the expanded LA thrombi from the RSPV thrombi, as determined by TEE. Moreover, this is the report of the amelioration of T2DM using standard-dose heparin-warfarin remedy. T2DM is an increasingly important disease, and many remedies, such as diet, exercise and medications, including insulin, are used to treat this disease. One month of standard-dose heparin-warfarin remedy might be a new remedy for patients with T2DM. Heparin is known to disrupt histones, which are major components of NETs, and reduce thrombosis [17-19], suggesting that heparin blocks microclots in microvessels, decreasing insulin resistance. Heparin might restore blood flow in microvessels. Moreover, diabetes nephropathy and gestational diabetes mellitus are clinically very important diseases. Diabetes nephropathy [13] and gestational diabetes mellitus [15] have been reported to be associated with NETs. Therefore, standard-dose heparin-warfarin remedy could ameliorate these diabetes-associated diseases. Low-molecular-weight heparin was reported to be useful for treating gestational diabetes mellitus [20]. The role of warfarin is unclear. More studies are needed to determine the additional mechanisms involved in these relationships.

## Conclusions

One month of standard-dose heparin-warfarin remedy partially resolved the RSPV thrombi and expanded LA thrombi from the RSPV thrombi. The patient’s RSPV thrombi and expanded LA thrombi from the RSPV thrombi were detected using cardiac CT. However, on TEE, these thrombi were depicted as black areas, and they moved periodically inward with the patient’s heartbeats. The remedy ameliorated T2DM, the effect of which could be maintained for the next five months by administering a standard dose of warfarin.
